# Association of serum 25-hydroxyvitamin D with metabolic syndrome and type 2 diabetes: a one sample Mendelian randomization study

**DOI:** 10.1186/s12877-021-02307-6

**Published:** 2021-06-29

**Authors:** Jing Xiao, Jingyi Lv, Shiyu Wang, Yang Zhou, Lunwen Chen, Juying Lu, Xiaoyi Zhang, Xiaojian Wang, Yunjuan Gu, Qingyun Lu

**Affiliations:** 1 Department of Epidemiology and Medical Statistics, School of Public Health, Nantong University, No.9 Seyuan Road, Chongchuan District, Nantong, Jiangsu P.R. China 226019; 2 Department of Endocrinology and Metabolism, Affiliated Hospital of Nantong University, No.20 Xisi Road, Chongchuan District, Nantong, Jiangsu P.R. China 226001; 3 Department of Chronic Disease and Prevention, Center for Disease Control and Prevention of Haian, Nantong, Jiangsu P.R. China 226600

**Keywords:** 25-hydroxyvitamin D, Genetic risk scores, Mendelian randomization, Metabolic syndrome, Type 2 diabetes

## Abstract

**Background:**

Vitamin D deficiency has been associated with type 2 diabetes (T2D) and metabolic syndrome (MS) and its components. However, it is unclear whether a low concentration of vitamin D is the cause or consequence of these health conditions. Thus, this study aimed to evaluate the association of vitamin D concentrations and its genetic risk scores (GRSs) with MS and its component diseases, such as T2D, in middle-aged and elderly participants from rural eastern China.

**Methods:**

A subset of 2393 middle-aged and elderly individuals were selected from 70,458 participants of the Nantong Chronic Diseases Study of 2017–2018 in China. We used two 25-hydroxyvitamin D (25[OH]D) synthesis single-nucleotide polymorphisms (SNPs) (DHCR7-rs12785878 and CYP2R1-rs10741657) and two 25(OH) D metabolism SNPs (GC-rs2282679 and CYP24A1-rs6013897) for creating GRSs, which were used as instrumental variables to assess the effect of genetically lowered 25(OH) D concentrations on MS and T2D based on the Wald ratio. F statistics were used to validate that the four SNPs genetically determined 25(OH) D concentrations.

**Results:**

Compared to vitamin D sufficient individuals, individuals with vitamin D insufficiency had an odds ratio (OR [95% confidence interval {CI}]) of MS of 1.30 (1.06–1.61) and of T2D of 1.32 (1.08–1.64), individuals with vitamin D deficiency had an ORs (95% CI) of MS of 1.50 (1.24–1.79) and of T2D of 1.47 (1.12–1.80), and those with vitamin D severe deficiency had an ORs (95% CI) of MS of 1.52 (1.29–1.85) and of T2D of 1.54 (1.27–1.85). Mendelian randomization analysis showed a 25-nmol/L decrease in genetically instrumented serum 25(OH) D concentrations using the two synthesis SNPs (DHCR7 and CYP2R1 genes) associated with the risk of T2D and abnormal diastolic blood pressure (DBP) with ORs of 1.10 (95%CI: 1.02–1.45) for T2D and 1.14 (95%CI: 1.03–1.43) for DBP.

**Conclusions:**

This one sample Mendelian randomization analysis shows genetic evidence for a causal role of lower 25(OH) D concentrations in promoting of T2D and abnormal DBP in middle-aged and elderly participants from rural China.

**Supplementary Information:**

The online version contains supplementary material available at 10.1186/s12877-021-02307-6.

## Background

Metabolic syndrome (MS) encompasses a cluster of conditions such as abdominal obesity, hypertension, dyslipidemia, and hyperglycemia [1], that contribute to an increased risk of diabetes, heart disease, and death [2]. MS is a serious burden on public health, and its management is difficult [3]. China and many other Asian countries have recently been experiencing dramatic increases in cases of MS and its components, leading to a high incidence of ensuing problems, especially in middle-aged and elderly Chinese populations [4–7]. During the period from 2014 to 2015, the prevalence of MS, type 2 diabetes (T2D), and hypertension were approximately 18.4, 8.5, and 36.6%, respectively, in the middle-aged Chinese population, and 22.8, 15.3, and 55.7%, respectively, in the elderly Chinese population [4, 6, 7]. The etiology of MS and its components involves a complex interaction of multiple genetic and environmental factors, and its suggested heritability estimates range from 13 to 30% [8, 9].

Vitamin D deficiency is common in European, Indian, South American, and Chinese populations and is particularly notable in middle-aged and elderly Chinese populations [10, 11]. Vitamin D deficiency is associated with MS [10], hypertension [12], cardiovascular disease [13], glucose homeostasis, and T2D [14], as well as obesity and abdominal obesity [15]. Serum 25-hydroxyvitamin D (25[OH]D), a generally accepted biomarker of circulating vitamin D levels in humans, has been found to be inversely associated with MS and T2D in middle-aged and elderly individuals from China [10, 16]. However, the rationale for low concentrations of vitamin D contributing to MS and its associated diseases remains unclear. Studies on genetic variants that specifically affect 25(OH) D concentrations may aid in clarifying the causal association.

Advances in the methodology of large-scale genetic association studies along with international collaboration have identified four single-nucleotide polymorphisms (SNPs) from four genes that influence 25(OH) D concentrations [17, 18]. Genetic variants of synthesis genes *DHCR7/NADSYN1* (7-dehydrocholesterol reductase) and *CYP2R1* (25-hydroxylase) affect the synthesis of 25(OH)D; the transport gene *GC* (group-specific component) encodes the vitamin D-binding protein, and the catabolism gene *CYP24A* (24-hydroxylase) is involved in the clearance of 25(OH) D [19].

We calculated genetic risk scores (GRSs) as instrumental variables (IVs) to estimate the causal effects of circulating vitamin D on MS and T2D; Mendelian randomization (MR), which refers to the random allocation of alleles during meiosis, was used [20]. In MR, allocation is expected to be independent of behavioral and environmental factors, thus allowing for the assessment of non-confounded risk associations that are not because of reverse causality [20, 21]. MR uses genetic variants as IVs to assess the causal effect of phenotypes, such as vitamin D status, on diseases such as MS, and this is believed to limit unmeasured confounding [21]. However, causal associations between vitamin D and metabolic diseases remain unclear. Previous studies have not provided consistent results [22–28]. It has been reported that every 10% increase in genetically instrumented 25(OH) D concentrations is associated with decreased diastolic blood pressure (DBP) and an 8.1% decrease in the risk of hypertension [25]. A 25-nmol/L higher genetically instrumented 25(OH) D concentration using two synthesis SNPs was associated with a 14% lower risk of T2D; conversely, no association was found between genetically instrumented 25(OH) D using four vitamin D-related SNPs and T2D [24]. However, other studies conducted in China have reported no association of genetically determined 25(OH) D concentrations with MS and its metabolic traits [23] or T2D [26]. Nevertheless, these studies did not specifically target middle-aged and elderly populations. Thus, this study aimed to evaluate the association between serum 25(OH) D concentrations and its genetic determinants with MS and its component diseases, such as T2D, in middle-aged and elderly participants from rural eastern China.

## Methods

### Participants and study design

The Nantong Chronic Diseases Study included a cohort of 70,458 participants, aged 18–90 years who were enrolled from six communities in Nantong, China between 2017 and 2018. A subset of 2393 middle-aged and elderly people (aged above 45 years) was selected for this study. Information on demographics, lifestyle, personal medical history, and family history of chronic diseases was collected by trained interviewers during an in-person interview; participants were asked to provide a fasting blood sample. The study protocol was approved by the Institutional Review Boards of Nantong University and the Nantong Centers for Disease Control. All participants provided written informed consent.

**Fig. 1:**
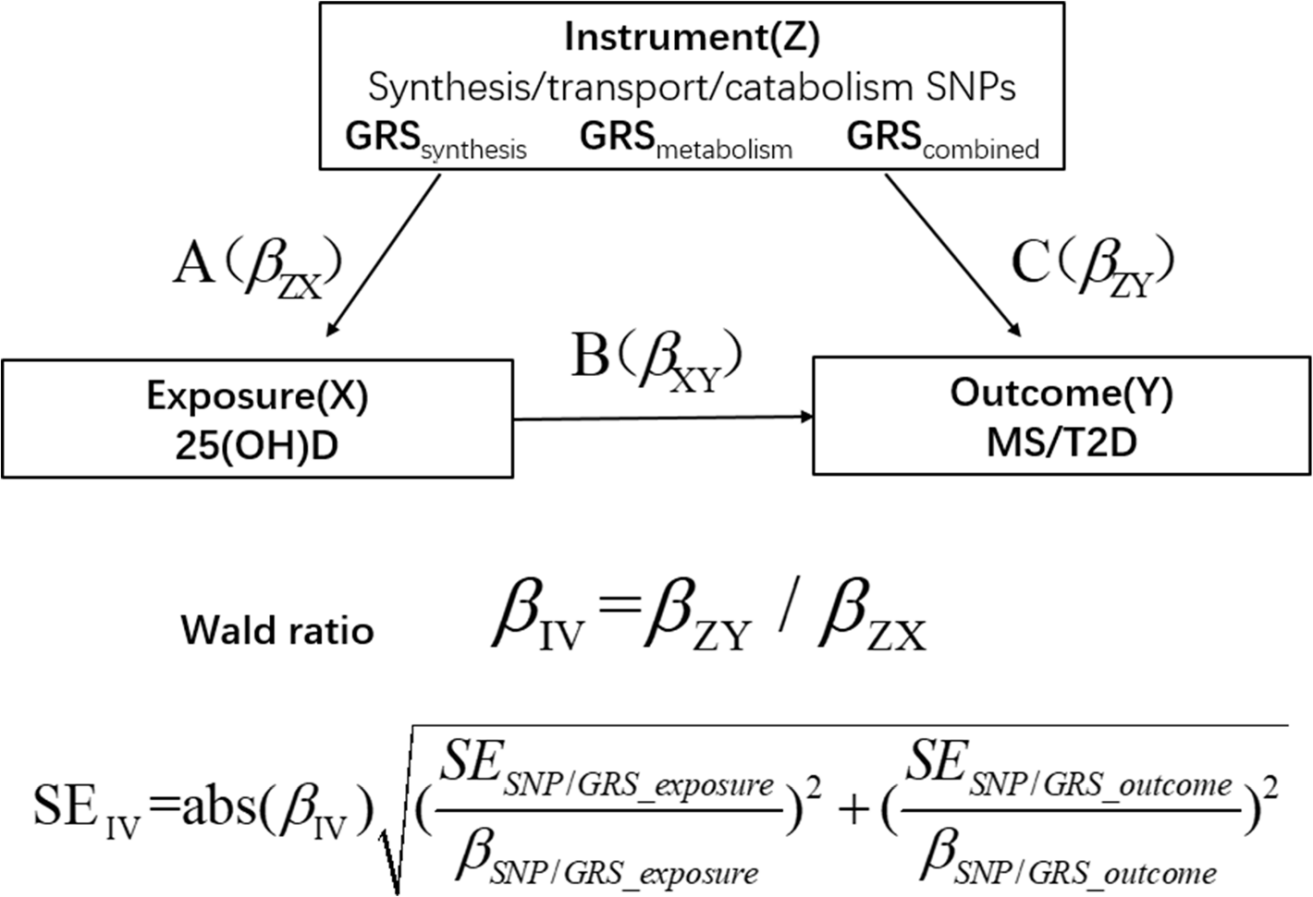
Study design and tested associations. GRS, genetic risk score; 25(OH) D, 25-hydroxyvitamin D; IV, instrumental variable

The study design is shown in Fig. 1. In this study, associations between 25(OH)D-related genetic variants and 25(OH) D concentrations (A(*β*_ZX_)) and between 25(OH)D-related genetic variants and MS/T2D (C(*β*_ZY_)) were assessed, and observational assessments of the association between 25(OH) D concentrations and MS/T2D (B(*β*_XY_)) were recorded.

### Anthropometric and biochemical measurements

Anthropometric measurements of weight, height, and waist and hip circumference (WC and HC, respectively) were performed twice according to a standard protocol. If the difference between the first two measurements was greater than 1 cm for circumference or 1 kg for weight, a third measurement was taken. The averages of the two closest measurements were then used in the present study. The waist-hip ratio (WHR) and body mass index (BMI) were calculated from these measurements; BMI was calculated as the weight in kg divided by the square of height in meters.

Serum 25(OH) D concentrations were assayed using enzyme linked immunosorbent assay; we defined 25(OH) D concentrations < 25 nmol/L as severe deficiency, 25 to < 50 nmol/L as deficiency, 50 to < 75 nmol/L as insufficiency, and ≥ 75 nmol/L as sufficiency [29]. Levels of fasting blood glucose (FBG) and blood lipids (triglyceride [TG] and high-density lipoprotein cholesterol [HDL-c]) were also measured; insulin levels were measured using a chemiluminescent immunoassay. The homeostasis model assessment of insulin resistance (HOMR-IR) was calculated based on the following formula: HOMR-IR = (FBG (mmol/L) × insulin (μIU/mL))/22.5. Blood pressure, comprising of both systolic blood pressure (SBP) and diastolic blood pressure (DBP), was assessed twice in a time interval of more than 3 min. If the difference between the first two measurements was larger than 10 mmHg, a third measurement was performed; the averages of the two closest measurements were used in this study. Other demographic data, including those related to education, income, lifestyle factors (i.e., physical activity, smoking and alcohol consumption status), personal medical history, family history of chronic diseases, and use of vitamin D and calcium supplements, were collected using a standardized questionnaire.

### Diagnostic criteria for MS and T2D

In this study, the definition of MS was based on the joint interim statement of the International Diabetes Federation criteria [30], adopting the Asian criteria for WC. It mandated the inclusion of ≥3 of the following metabolic abnormalities: central obesity: WC ≥85 cm for Chinese men and ≥ 80 cm for Chinese women; abnormal fasting blood serum TG levels ≥1.7 mmol/L or taking TG-lowering medication; abnormal fasting serum HDL-c levels < 1.3 mmol/L for Chinese women and < 1.0 mmol/L for Chinese men or receiving treatment for increasing HDL-c levels; abnormal blood pressure with SBP ≥ 130 mmHg, DBP ≥ 85 mmHg, or receiving antihypertensive medication; and abnormal fasting serum glucose (prediabetes) level ≥ 5.6 mmol/L or receiving anti-diabetic medication.

T2D was defined based on FBG levels ≥7.0 mmol/L and/or 2-h oral glucose tolerance test results ≥11.1 mmol/L; treatment with anti-diabetic medication and/or previous diagnosis of diabetes by a physician were also used to define the presence of T2D [31].

### SNP selection and genotyping

For the four vitamin D-related SNPs, two synthesis SNPs (*DHCR7/NADSYN1*-rs12785878 and *CYP2R1*-rs10741657), one transport SNP (*GC-*rs2282679), and one catabolism SNP (*CYP24A1*-rs6013897) were selected; this is similar to the method of a recent study in an Asian cohort [32]. These SNPs were significantly associated with plasma 25(OH) D concentrations in a previous genome-wide association study (GWAS) [18] and were also used in Mendelian analyses in studies on Chinese populations [23, 24]. The genotyping concordance was > 99.9%, and the genotype success rate was 99.9% for each SNP. All four SNPs were on the Hardy-Weinberg equilibrium (*P* > 0.05), and the frequency of alleles was > 0.05.

Genotyping was performed using the iPLEX™ Sequenom MassARRAY® platform. Polymerase chain reaction (PCR) and extension primers were designed using MassARRAY Assay Design 3.0 software (Sequenom, Inc). PCR and extension reactions were performed according to the manufacturer’s instructions, and extension product sizes were determined using mass spectrometry with the Sequenom iPLEX system. On each 96-well plate, two negative controls (water), two blinded duplicates, and two samples were included.

### GRSs

We assumed an additive genetic model for SNPs with scores of 0, 1, or 2 for genotypes containing 0, 1, or 2 alleles, respectively, based on the relationship between the SNPs and circulating vitamin D levels. GRSs were the sum of scores for each SNP multiplied by the *β* value from a previous study [32]. We calculated the GRS_synthesis_ for two synthesis SNPs, (*DHCR7*-rs12785878 *+ CYP2R1*-rs10741657), GRS_metabolism_ for two metabolism SNPs (*GC-*rs2282679 + *CYP24A1*- rs6013897), and GRS_combined_ for all four SNPs.

### Statistical analyses

Normally distributed continuous variables are presented as means ± standard deviation (SD) and compared using ANOVA. Non-normally distributed continuous variables are expressed as medians (interquartile range [IQR]) and analyzed using the Wilcoxon rank sum test. Categorical variables are expressed as percentages; the Pearson’s chi-square test was used for comparison between MS/T2D cases and MS/T2D non-cases. Furthermore, clinical characteristics (such as glucose, TG, hypertension, among others) were compared between the quintiles of 25(OH) D concentration groups using ANOVA for continuous variables with normal distribution, the Kruskal-Wallis test for continuous variables with non-normal distribution, and Pearson’s chi-square test for categorical variables.

First, linear regression was used to determine the association of each SNP with 25(OH) D concentrations (A(*β*_ZX_)), assuming linear effects of each vitamin-D related SNP per additional allele on 25(OH)D. The Cragg-Donald F-statistic $\left[F=\frac{\left({R}^2\ast \left(\mathrm{n}\hbox{-} 2\right)\right)}{\left(1-{R}^2\right)}\right]$ was used to estimate the strength of the association, and F values > 10 were regarded as useful for MR analysis [33]. The association of each SNP with MS and T2D was then determined using logistic regression (C(*β*_ZY_)). Genetically determined A(*β*_ZX_) and C(*β*_ZY_) were calculated using the Wald ratio estimator (*β*_IV_ = *β*_ZY_/*β*_ZX_) [34]. The Wald ratios of the four SNPs were meta-analyzed in an inverse-variance weighted meta-analysis to compute the MR estimate based on the fixed effect model ${\hat{\beta}}_{\mathrm{IVW}}=\left(\sum \limits_{j=1}^4{\omega}_j{\beta}_{IV_j}\right)/\sum \limits_{j=1}^4{\omega}_j$, ${\omega}_j=1/\operatorname{var}\left({\beta}_{IV_j}\right),\kern0.5em j=1,\dots \dots, 4$. Furthermore, the effects of pleiotropy for the four SNPs were analyzed using MR-Egger regression, in which the *p* value of intercept gives a valid test of directional pleiotropy [35].

To explore the observational associations (*β*_XY_) of 25(OH) D with MS/T2D, logistic regression analysis was used to estimate the odds ratios (ORs) and 95% confidence intervals (CIs). Effect estimates were presented per 10-nmol/L or per 25-nmol/L decrease in 25(OH) D concentrations or quintiles 25(OH) D adjusted for confounders, that differed between MS/T2D cases and non-MS/T2D cases.

The association of GRS_synthesis_, GRS_metabolism_, and GRS_combined_ with 25(OH) D (*β*_*GRS* − *VD*_) was estimated using linear regression analysis. GRS_combined_, GRS_synthesis_, and GRS_metabolism_ were used as IVs to estimate the causal effect of 25(OH) D on T2D and MS and its components adjusted for confounders. IV estimates of genetically determined OR were obtained with the Wald-type estimator *OR*_*IV*(*VD* − *outcome*)_ =  *exp* (ln(*OR*_*GRS* − *outcome*_)/*β*_*GRS* − *VD*_). Effect estimates of GRSs have been presented per unit higher in GRS; *OR*_*IV*(*VD* − *outcome*)_ denoted a genetically determined of per 25-nmol/L decrease in 25(OH) D concentrations.

Another method for calculating IV employs a two-stage regression estimator to calculate causal ORs per 25-nmol/L increase in 25(OH) D concentrations to explain the sensitivity analyses [36]. In the first stage, a linear regression of 25(OH) D on GRSs was used to generate 25(OH)D-fitted values. In the second stage, the predicted 25(OH) D values from the first stage were used for logistic regression with T2D and MS and its components as the dependent variable.

All analyses were performed using SAS (version 9.3; SAS Institute, Cary, NC), and *P* < 0.05 was considered statistically significant; all results were based on two-sided probability.

## Results

The prevalence of MS and T2D in the 2393 participants were 31.17 and 15.09%, respectively. Table 1 presents the differences among select demographic characteristics, anthropometric measurements, and lifestyle factors between MS/T2D cases and MS/T2D non-cases. Both MS and T2D cases were older, had higher weight, greater WC, higher BMI, and greater WHR than non-MS/T2D cases. The composition ratio of MS/T2D and non-MS/T2D cases was not balanced among the groups based on different income, alcohol consumption status, physical activity, or familial history of MS/T2D. Moreover, the composition ratio of MS and non-MS cases was not balanced with regard to smoking status.

**Table 1:** Characteristics of study participants: MS/T2D cases and non-cases (*n* = 2393)

	MS cases (*n* = 746)	MS non-cases (*n* = 1647)	P	T2D cases (*n* = 361)	T2D non-cases (*n* = 2032)	P
Age (year, $\overline{x}\pm SD$)	61.24 ± 6.41	56.68 ± 6.19	< 0.001^*^	60.30 ± 6.54	57.71 ± 6.41	< 0.001^*^
Weight (kg, $\overline{x}\pm SD$) ^1^	64.35 ± 18.41	58.85 ± 18.20	< 0.001^*^	66.26 ± 18.92	54.87 ± 18.63	< 0.001^*^
WC (cm, $\overline{x}\pm SD$) ^1^	87.76 ± 10.32	78.75 ± 10.16	< 0.001^*^	89.86 ± 11.26	76.10 ± 10.97	< 0.001^*^
BMI (kg/m^2^, $\overline{x}\pm SD$) ^1^	26.66 ± 4.02	22.47 ± 3.87	< 0.001^*^	27.62 ± 4.12	21.38 ± 4.06	< 0.001^*^
WHR ($\overline{x}\pm SD$)^1^	0.94 ± 0.14	0.88 ± 0.12	< 0.001^*^	0.95 ± 0.13	0.87 ± 0.11	< 0.001^*^
Education (%)^1^			0.259			0.644
Illiterate	0.00	100.00		0.00	100.00	
Primary school	28.48	71.52		12.06	87.94	
Middle school	30.23	69.77		14.86	85.14	
High school	32.26	67.74		15.25	84.75	
Colleges and above	31.86	68.14		15.99	84.01	
Income (yuan/month, %)^1^			< 0.001^*^			0.040^*^
< 2000	26.53	73.47		13.01	86.99	
2000-	30.49	69.51		14.33	85.67	
3500-	31.32	68.68		15.64	84.36	
≥ 3500	43.61	56.39		21.10	78.90	
Smoking status (%)^1^			< 0.001^*^			0.488
Never-smokers	26.90	73.10		14.72	85.28	
Ever smokers	32.69	67.31		13.70	86.30	
Current smokers	38.89	61.11		16.33	83.67	
Drinking status (%)^1^			< 0.001^*^			0.003^*^
Never-drinkers	28.87	71.13		14.35	85.65	
Ever drinkers	47.36	52.64		27.68	72.32	
Current drinkers	33.18	66.82		14.98	85.02	
Physical activity (%)^1^			< 0.001^*^			0.042^*^
No	33.00	67.00		15.89	84.11	
Yes	24.86	75.14		12.31	87.69	
CHD (%)^1^			0.352			0.729
No	31.01	68.99		15.03	84.97	
Yes	37.42	62.58		17.43	82.57	
Familial history of MS/T2D (%)			< 0.001^*^			0.040^*^
No	28.29	71.71		14.40	85.60	
Yes	44.55	55.45		18.25	81.75	
Vitamin D supplement (%)			0.830			0.849
No	31.22	68.78		15.09	84.91	
Yes	29.64	70.36		14.78	85.22	
Calcium supplement (%)			0.585			0.739
No	31.27	68.73		15.04	84.96	
Yes	28.88	71.12		16.15	83.85	

Abbreviations: *WC* waist circumference, *BMI* body mass index, *WHR* waist-hip ratio, *CHD* coronary heart disease, ^1^: adjusted for age at interview. ^*^: P < 0.05. ANOVA was performed on age at interview, weight, WC, BMI and WHR; the χ^2^ test was performed on the other variables to compare differences between case and non-case groups

We found significant differences in demographic and clinical characteristics among the five quintile groups of serum 25(OH) D concentrations (Table 2). The median FBG (5.49 mmol/L), insulin (73.24 pmol/L), HOMA-IR (1.41), and TG (0.97 mmol/L) values and the mean WC (75.26 cm) were the lowest, while the median HDL-c (1.35 mmol/L) value was the highest in the highest quintile (Q5) of serum 25(OH)D.

**Table 2:** Comparison of clinical characteristics among different groups based on serum 25(OH) D concentration

	Vitamin D Concentration (nmol/L)					P
	Q1(< 28.4)	Q2(28.5–36.7)	Q3(36.8–45.9)	Q4(46.0–57.4)	Q5(≥57.5)	
n	476	483	495	468	471	
Age (year, $\overline{x}\pm SD$)	59.81 ± 7.04	59.58 ± 7.02	59.97 ± 6.94	59.44 ± 7.18	58.82 ± 7.07	0.070
Female, n (%)	291(61.13)	285(59.00)	301(60.81)	282(60.26)	279(59.24)	0.950
FBG (mmol/L, M (IQU))	5.80(1.09)	5.69(1.19)	5.68(1.19)	5.60(1.08)	5.49(0.90)	0.002^*^
Insulin (pmol/L, M (IQU))	88.24(61.70)	87.96(60.38)	83.15(52.22)	80.78(47.42)	73.24(43.8)	< 0.001^*^
HOMA-IR	1.69(1.12)	1.68(1.09)	1.60(0.94)	1.50(0.88)	1.41(0.82)	< 0.001^*^
TG (mmol/L, M (IQU))	1.24(0.97)	1.23(0.98)	1.20(0.98)	1.09(0.88)	0.97(0.74)	< 0.001^*^
HDL-c (mmol/L, M (IQU))	1.21(0.99)	1.24(0.98)	1.24(0.98)	1.28(1.00)	1.35(0.96)	< 0.001^*^
BMI (Kg/m^2^, $\overline{x}\pm SD$)	25.15 ± 3.98	24.92 ± 4.01	25.18 ± 3.87	24.64 ± 3.77	24.44 ± 4.05	0.064
WC (cm, $\overline{x}\pm SD$)	86.33 ± 11.09	85.83 ± 10.52	84.86 ± 10.75	81.45 ± 11.04	75.26 ± 10.43	< 0.001^*^
Hypertension, n (%)	267(56.09)	271(56.11)	273(55.15)	240(51.28)	230(48.83)	0.085
Familial history of diabetes, n (%)	69(14.50)	73(15.11)	72(14.55)	60(12.82)	59(12.53)	0.717
Familial history of CHD, n (%)	120(25.21)	119(24.64)	122(24.65)	117(25.00)	102(21.66)	0.701

^*^: P < 0.05. *Abbreviations* BMI body mass index, *CHD* coronary heart disease, *FBG* fasting blood glucose, *HDL-c* high-density lipoprotein cholesterol, *HOMA-IR* homeostasis model assessment of insulin resistance, *TG* triglyceride, *WC* waist circumference, x¯±SD: mean ± standard deviation; M (IQU): median (interquartile range)

**Fig. 2:**
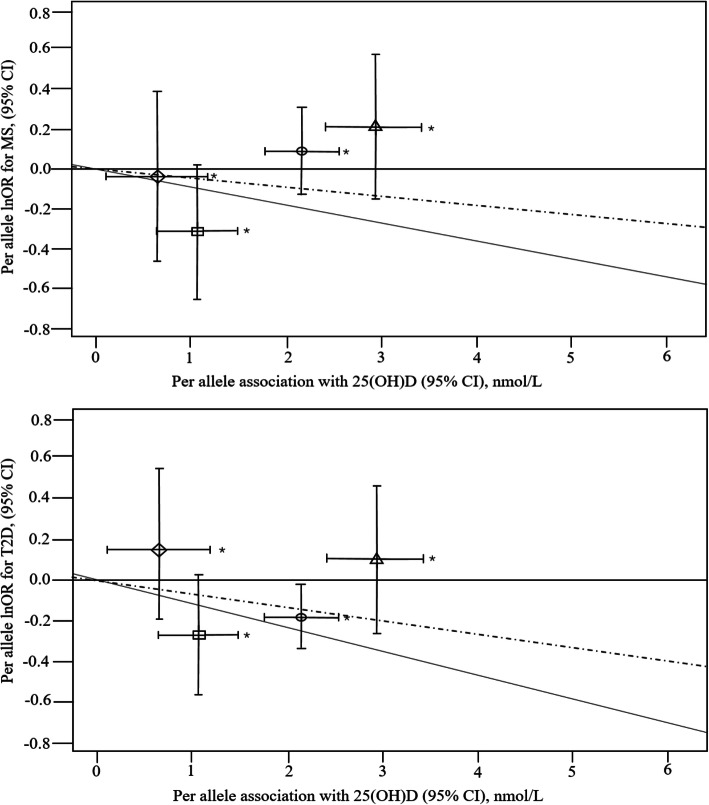
Scatterplot of associations of per allele effects of the four SNPs with risk of MS/T2D. This association was evaluated for 25(OH) D concentrations in our population based on the effect of the SNPs on 25(OH) D concentration (Square: CYP2R1 − rs10741657; Circle: DHCR7 − rs12785878; Triangle: GC/DBP − rs2282679; Diamond: CYP24A1 − rs6013897). ^*^: *P* < 0.05. Dashed line: The slope from an inverse-variance weighted linear regression of per allele lnOR for MS/T2D for all four SNPs on 25(OH) D concentrations, with the line forced through the origin. Fulled line: The slope from an inverse-variance weighted linear regression of per allele lnOR for MS/T2D for two synthesis SNPs on 25(OH) D concentrations, with the line forced through the origin

Figure 2 shows the scatterplot of the associations of per allele effects of the four SNPs for 25(OH) D concentrations with the risk of MS/T2D, by their effects on 25(OH) D according to the study population. All four SNPs were associated with 25(OH) D concentrations (*P* < 0.05). Concentrations of 25(OH) D per allele (increasing 25(OH) D concentrations) were higher by 1.10 nmol/L (95% CI: 0.74–1.46 nmol/L) for CY2R1 rs10741657, 2.14 nmol/L (1.82–2.46 nmol/L) for DHCR7 rs12785878, 2.94 nmol/L (2.56–3.32 nmol/L) for GC rs2282679, and 0.74 nmol/L (0.32–1.16 nmol/L) for CYP24A1 rs6013897. The F-statistic ranged from 17.4 to 67.8 across each SNP, indicating an adequately strong association for MR analysis. However, none of the 25(OH)D-related SNPs were significantly associated with MS/T2D. The four SNPs accounted for the variation in exposure considerably more than the outcomes. The absolutes of the Wald ratios (95% CI) ranged from 0.02 (− 0.11–0.14) to 0.28 (0.01–0.86), indicating a causal effect of exposure (25(OH)D) on outcomes (MS/T2D). Moreover, Fig. 2 shows some evidence of pleiotropy for transport and catabolism SNPs, the *p*-value for the MR-Egger intercept was 0.043 and 0.052 for the four SNPs in T2D and MS, respectively, while the intercepts of two synthesis SNPs are almost to zero, suggested possible pleiotropy for metabolism SNPs.

Table 3 presents the association of serum 25(OH) D concentration with MS and T2D in the study population. Significant associations were found between serum 25(OH) D and both MS and T2D. Compared to the highest quintile of serum 25(OH) D (≥ 57.5 nmol/L), individuals with the third quintile of serum 25(OH) D (36.8–45.9 nmol/L) had an OR (95% CI) of MS of 1.29 (1.03–1.75) and of T2D of 1.33 (1.05–1.67), individuals with the fourth quintile of 25(OH) D (28.5–36.7 nmol/L) had an OR (95% CI) of MS of 1.47 (1.17–1.81) and of T2D of 1.50 (1.17–1.82), and individuals with the lowest quintile of serum 25(OH) D (< 28.4 nmol/L) had an OR (95% CI) of MS of 1.55 (1.24–1.89) and of T2D of 1.53 (1.21–1.87), respectively, among middle-aged and elderly Chinese participants. Similarly, compared to the sufficiency category of vitamin D (≥75 nmol/L), there was an increased prevalence of MS and T2D in the insufficiency category of vitamin D (25(OH)D: 50 to < 75 nmol/L), with ORs (95% CI) of 1.30 (1.06–1.61) and 1.32 (1.08–1.64), respectively; these values were 1.50 (1.24–1.79) and 1.47 (1.12–1.80), respectively, in the deficiency category of vitamin D (25(OH)D: 25 to < 50 nmol/L) and 1.52 (1.29–1.85) and 1.54 (1.27–1.85), respectively, in the severe deficiency category of vitamin D (25(OH)D: < 25 nmol/L). Overall, every 25-nmol/L decrease in serum 25(OH) D concentrations was associated with an increased risk of MS and T2D (OR [95% CI]: 1.23 [1.06–1.52] and 1.14 [1.05–1.33], respectively).

**Table 3:** Association of T2D and MS with serum 25 (OH) D concentration

	MS cases n (%)	OR_XY_ (95%CI)^1^	P	T2D cases n (%)	OR_XY_ (95%CI) ^2^	P
Every decreasing 10 nmol/L 25(OH)D	746(31.17)	1.10(1.02–1.23)	0.027^*^	361(15.09)	1.07(1.01–1.21)	0.018^*^
Adj-R^2^		0.732			0.756	
Every decreasing 25 nmol/L 25(OH)D		1.23(1.06–1.52)	0.002^*^		1.14(1.05–1.33)	0.001^*^
Adj-R^2^		0.789			0.764	
Quintiles of 25(OH) D (nmol/L)						
Q1(≥57.5)	135(26.68)	1.00		53(11.08)	1.00	
Q2(46.0–57.4)	129(28.48)	1.09(1.01–1.45)	0.025^*^	64(13.77)	1.12(1.03–1.59)	0.004^*^
Q3((36.8–45.9)	161(31.69)	1.29(1.03–1.75)	< 0.001^*^	75(15.14)	1.33(1.05–1.67)	< 0.001^*^
Q4(28.5–36.7)	167(34.08)	1.47(1.17–1.81)	< 0.001^*^	82(17.13)	1.50(1.17–1.82)	< 0.001^*^
Q5(< 28.4)	154(35.32)	1.55(1.24–1.89)	< 0.001^*^	87(18.29)	1.53(1.21–1.87)	< 0.001^*^
Adj-R^2^		0.784			0.753	
Clinical categories of 25(OH) D (nmol/L)						
≥ 75	21(26.12)	1.00		10(12.44)	1.00	
50–75	196(27.90)	1.30(1.06–1.61)	0.008^*^	92(13.08)	1.32(1.08–1.64)	0.001^*^
25–50	390(31.90)	1.50(1.24–1.79)	< 0.001^*^	188(15.41)	1.47(1.12–1.80)	< 0.001^*^
< 25	139(35.91)	1.52(1.29–1.85)	< 0.001^*^	71(18.25)	1.54(1.27–1.85)	< 0.001^*^
Adj-R^2^		0.713			0.762	

^1^adjusted for age at interview, *BMI, WHR*, income, smoking status, alcohol consumption status, physical activity, and familial history of MS; ^2^adjusted for age at interview, *BMI WHR*, income, alcohol consumption status, physical activity, and familial history of diabetes. ^*^: P < 0.05

Table 4 shows the causal coefficients from the MR analysis for the association of MS, T2D, and abnormal SBP and DBP with vitamin D-determined GRSs. Regarding OR_ZY_, one unit higher GRS_synthesis_ was associated with an increased risk of T2D and abnormal DBP (OR [95%CI]: 1.07 [1.01–1.42] and 1.16 [1.02–1.65], respectively). No significant associations were observed among GRS_metabolism,_ GRS_combined_, and T2D and MS and its components. Furthermore, no significant association was found between serum 25(OH)D-determining genetic variants and MS risk. However, MR analysis found that a per 25-nmol/L decrease in genetically instrumented serum 25(OH) D concentrations using two synthesis SNPs (*DHCR7*-rs12785878 + *CYP2R1*-rs10741657) was associated with an increasing risk of T2D (OR [95%CI]: 1.10 [1.02–1.45]) and abnormal DBP (1.14 [1.03–1.43]). Moreover, we did not find any association between genetically instrumented serum 25(OH) D concentration of two metabolism and all four SNPs with T2D. Null results were obtained between genetically instrumented serum 25(OH) D concentrations using any single SNP and MS or T2D in the middle-aged and elderly participants from eastern rural China (data not shown).

**Table 4:** Causal coefficients from MR analysis

GRSs	OR (95%CI) for MS	P	OR (95%CI) for T2D	P	OR (95%CI) for abnormal SBP	P	OR (95%CI) for abnormal DBP	P
GRS_synthesis_								
OR_ZY_ [per 1 unit higher in GRS]	1.21(0.88–1.66)	0.497	1.07(1.01–1.42)	0.048^*^	1.35(0.85–1.84)	0.435	1.16(1.02–1.65)	0.003^*^
OR_IV_ [per 25 nmol/L decrease 25(OH) D concentration]	0.85(0.56–1.28)	0.586	1.10(1.02–1.45)	0.014^*^	0.76(0.51–1.31)	0.141	1.14(1.03–1.43)	0.019^*^
GRS_metabolism_								
OR_ZY_ [per 1 unit higher in GRS]	1.06(0.79–1.44)	0.337	0.97(0.66–1.43)	0.646	0.92(0.73–1.17)	0.652	1.13(0.86–1.48)	0.627
OR_IV_ [per 25 nmol/L decrease 25(OH) D concentration]	0.92(0.69–1.22)	0.159	0.91(0.60–1.36)	0.742	1.04(0.74–1.45)	0.371	0.83(0.62–1.10)	0.579
GRS_combined_								
OR_ZY_ [per 1 unit higher in GRS]	1.05(0.83–1.32)	0.224	1.16(0.67–1.51)	0.154	0.79(0.60–1.02)	0.743	1.03(0.73–1.15)	0.085
OR_IV_ [per 25 nmol/L decrease 25(OH) D concentration]	1.04(0.70–1.53)	0.457	0.92(0.70–1.02)	0.082	0.88(0.67–1.15)	0.628	1.05(1.00–1.49)	0.046^*^

Adjusted for age at interview, BMI, WHR, income, smoking status, alcohol consumption status, physical activity and familial history of diabetes. ^*^: P < 0.05

The association of T2D, MS, and abnormal SBP and DBP with vitamin D-determined GRSs are shown

The results of the sensitivity analyses of another two-stage regression estimator for calculating the OR_IV_ per 25-nmol/L increase in 25(OH) D concentrations has been shown in Additional file 1. The results were considerably similar to those obtained using the Wald-type estimator. Significant associations were observed between 25(OH) D genetically determined by GRS_synthesis_ and T2D and abnormal DBP (OR [95%CI]: 1.08[1.03–1.38] and 1.09[1.02–1.37] per 25-nmol/L 25(OH) D decrease).

## Discussion

Vitamin D levels are known to influence the development of MS and component diseases, but the causality or direction of the association has been uncertain. This study showed that genetically determined 25(OH) D levels are causally associated with T2D and abnormal DBP in middle-aged and elderly rural participants from east rural China.

Many epidemiological studies have found inverse associations between serum 25(OH) D levels and MS and its component diseases [10, 37, 38]. Previous studies reported a positive correlation between vitamin D and HDL-c levels, but an inverse association with TG, SBP and DBP [10], T2D [38], BMI, and WC [39]. This study found that a higher serum 25(OH) D concentration was significantly associated with lower glucose concentrations, insulin levels, HOMA-IR, WC and higher HDL-c levels. Fully adjusted ORs (95% CI) for an increased risk of MS and T2D were 1.55 (1.24–1.89) and 1.53 (1.21–1.87) in the lowest quintile of serum 25(OH) D concentrations compared with the highest quintile of serum concentrations. This finding is consistent with the findings of the study by Afzal et al. [40] where the multivariable adjusted hazard ratio of T2D was 1.35 (1.09–1.66) for the lowest quartile compared with the highest quartile of serum 25(OH)D. The finding is also consistent with the findings of Bea et al. [37], in that serum 25(OH) D in the highest quartile decreased the risk for MS (OR = 0.52 [0.36–0.75]) compared with the lowest quartile of 25(OH)D. However, a number of randomized controlled trials have shown no association between vitamin D levels and the incidence of MS and its component diseases, including T2D in elderly people [41–43]. Furthermore, a cohort study reported that after a year of vitamin D supplementation, people whose serum 25(OH) D concentrations improved to < 25 nmol/L, 25 to < 50 nmol/L, 50 to < 75 nmol/L, and ≥ 75 nmol/L had a 0.76-, 0.64-, 0.59-, 0.56-fold risk, respectively, for MS at follow-up [44].

MR studies have provided no evidence to suggest that genetically increased serum 25(OH) D concentrations are associated with a lower risk of MS, T2D, or hypertension [23, 27, 45]. The current study did not identify any association of genetically determined vitamin D concentrations, associated SNPs, and GRSs with the risk of MS; however, it did show that a genetically instrumented 25-nmol/L decrease in serum 25(OH) D concentrations using two synthesis SNPs was associated with a 10% higher risk of T2D in these middle-aged and elderly participants from eastern rural China. The findings are consistent with those from the study by Lu et al. who found a 9% higher risk of diabetes in Chinese participants and a 14% higher risk of diabetes from a meta-analysis [24]. Furthermore, a study by Yuan et al. revealed that genetic variants associated with low plasma concentrations were associated with T2D (*P* = 0.0290) [28]. A previous study reported a modest association between the genetic scores of two SNPs of plasma 25(OH) D concentrations and hypertension (*P* = 0.0003) [25]; however, another investigation found no effect on blood pressure in a Chinese population [24]. The present study found that genetically instrumented 25-nmol/L decreases in serum 25(OH) D concentrations using two synthesis SNPs was associated with a 14% higher risk of abnormal DBP in these middle-aged and elderly participants from eastern rural China. It is known that the vitamin D synthesis genes *DHCR7*/*NADSYN1* and *CYP2R1*, transport gene *GC*, and catabolism gene *CYP24A1* contribute to variability in circulating biomarkers of vitamin D levels [46, 47]. Interestingly, GRSs combined with two metabolism SNPs or four SNPs in these four vitamin D-associated genes demonstrated no association with T2D and SBP/DBP. However, several studies have reported that both transport and catabolism SNPs show pleiotropy; this includes cases where the MR-Egger regression was used for meta-analysis [24, 47, 48]. Analysis of the association of transport and catabolism SNPs with MS/T2D also shows modest statistically significant pleiotropy in this study. Previous studies have suggested biological evidence of pleiotropy for GC/DBP-rs2282679, which influences vitamin D-binding protein; this results in discrepancies in the ratios of free to total plasma 25(OH) D concentrations and alterations in the feedback control of 25(OH) D concentrations [48, 49]. In addition, the vitamin D-binding protein also carries actin, a chemotactic factor implicated in inflammation, that may affect diabetes independent of effects on 25(OH) D concentrations [50].

Using a genetic variant as a proxy for vitamin D levels has been considered to provide better causal inferences for several reasons. First, unlike vitamin D levels, genetic variants are generally not associated with behavioral, social, and physiological factors that confound the association between vitamin D and MS and its associated diseases. Second, genetic variants associated with vitamin D levels are not influenced by other diseases, and the estimates are therefore less biased. Third, a genetic variant will often reflect exposure throughout the life course and will not change with disease status [51–53]. Finally, using multiple SNPs in different gene loci to index vitamin D levels, this study could minimize the risk of pleiotropic effects; this may be attributed to the fact that the effects of alternative pathways reflected by individual SNPs were strongly diluted when combined in a multi-marker score [54].

A limitation of the present study was the single measurement of vitamin D levels. The lack of 25(OH) D GWAS in Asian populations that would allow building of an ethnic-specific genetic score limits the use of MR. Furthermore, the four SNPs explain only approximately 1–4% of the variance in the 25(OH) D phenotype [18]. A GRS composed of a larger number of SNPs instrumental for vitamin D levels needs to be calculated to allow for precise estimation based on larger GWAS consortia [55]. Although MR is a potentially powerful technique for strengthening causal inference, several issues could disturb instrumental variable assumptions; these include developmental changes compensating for genetic variation; linkage disequilibrium between genotype and other causal variables; pleiotropy, which refers to a single gene having multiple biological functions [56]; and epigenetic effects, i.e., non-Mendelian heritable changes in gene expression not accompanied by changes in DNA sequence [21, 57]. This analysis is based on the assumption that the genotype only affects MS and its associated diseases through vitamin D levels.

## Conclusions

The serum 25(OH) D concentration was inversely associated with MS and T2D risk in the rural middle-aged and elderly participants. However, MR analysis demonstrated concordance between genetically determined 25(OH) D using two synthesis SNPs and the risk of T2D and abnormal DBP in middle-aged and elderly participants from eastern rural China. This indicates a risk effect of lower serum 25(OH) D concentrations on the development of T2D and abnormal DBP. Conversely, genetically determined vitamin D was not significantly associated with the development of MS; lower vitamin D concentrations are therefore unlikely to have a causal role in the development of MS. Therefore, further trials will be required on vitamin D supplementation before advocating the use of vitamin D supplements or food fortification for the prevention of MS and T2D.

## Supplementary Information

**Additional file 1.** Causal coefficients from MR analysis.

## Data Availability

The data sets analyzed in this study are available from the corresponding author upon reasonable request.

## Abbreviations

BMI: Body mass index
CI: Confidence interval
DBP: Diastolic blood pressure
FBG: Fasting blood glucose
GRS: Genetic risk scores
GWAS: Genome-wide association study
HDL-c: High-density lipoprotein cholesterol
HOMR-IR: Homeostasis model assessment of insulin resistance
IQR: Interquartile range
MR: Mendelian randomization
MS: Metabolic syndrome
ORs: Odds ratios
PCR: Polymerase chain reaction
SBP: Systolic blood pressure
SD: Standard deviation
SNP: Single-nucleotide polymorphism
T2D: Type 2 diabetes
TG: Triglyceride
WC: Waist circumference
WHR: Waist-hip ratio
