# Supplementary material for: Association of serum 25-hydroxyvitamin D with metabolic syndrome and type 2 diabetes: a one sample Mendelian randomization study
Source: BMC Geriatr. 2021 Jun 29;21:391. doi: 10.1186/s12877-021-02307-6 (PMC8244233; doi:10.1186/s12877-021-02307-6)
Supplement: Supplementary file 1 — Additional file 1. Causal coefficients from MR analysis. [file 12877_2021_2307_MOESM1_ESM.doc]

**Additional file 1. Causal coefficients from MR analysis**

| GRSs | OR (95%CI)  for MS | P | OR (95%CI)  for T2D | P | OR (95%CI)  for abnormal SBP | P | OR (95%CI)  for abnormal DBP | P |
| --- | --- | --- | --- | --- | --- | --- | --- | --- |
| GRS_synthesis_ |  |  |  |  |  |  |  |  |
| OR_IV_[per 25nmol/L decrease 25(OH)D concentration] | 1.13(0.71-1.45) | 0.207 | 1.08(1.03-1.38) | **0.031**^*^ | 1.03(0.74-1.27) | 0.314 | 1.09(1.02-1.37) | **0.018**^*^ |
| GRS_metabolism_ |  |  |  |  |  |  |  |  |
| OR_IV_[per 25nmol/L decrease 25(OH)D concentration] | 0.98(0.74-1.30) | 0.274 | 1.04(0.73-1.27) | 0.631 | 0.89(0.57-1.38) | 0.326 | 1.01(0.72-1.40) | 0.282 |
| GRS_combined_ |  |  |  |  |  |  |  |  |
| OR_IV_[per 25nmol/L decrease 25(OH)D concentration] | 1.02(0.72-1.43) | 0.317 | 1.07(0.98-1.30) | 0.057 | 0.91(0.59-1.42) | 0.173 | 1.04(1.01-1.38) | 0.052 |

Adjusted for age at interview, BMI, WHR, income, smoking status, alcohol consumption status, physical activity and familial history of diabetes. ^*^: P<0.05.

The association of T2D, MS, and abnormal SBP and DBP with vitamin D-determined GRSs are shown.
